# What are the factors associated with sarcopenia-related variables in adult women with severe obesity?

**DOI:** 10.1186/s13690-020-00454-7

**Published:** 2020-08-03

**Authors:** Erika Aparecida Silveira, Jacqueline Danesio de Souza, Annelisa Silva e Alves de Carvalho Santos, Andrea Batista de Souza Canheta, Valéria Pagotto, Matias Noll

**Affiliations:** 1 Health Science Post-Graduation Program, Faculty of Medicine, Universidade Federal de Goiás, 1a. s/n - Setor Leste Universitário, Goiânia, Goiás CEP 74605-020 Brazil; 2 University North of Paraná, Londrina, Brazil; 3 Faculty of Nursing, Universidade Federal de Goiás, Goiânia, Brazil; 4 Instituto Federal Goiano, Goiânia, Brazil

**Keywords:** Sarcopenic obesity, muscle mass, Muscle strength, Walking speed, metabolic diseases

## Abstract

**Background:**

Understanding the association between sarcopenia-related variables and several risk factors may help to implement interventions aimed at preventing its occurrence by reducing or controlling the identified risk factors. Although changes in body composition occur in both sexes, in women, muscle loss is accentuated due to decreased estrogen levels following menopause. This study aims to determine the factors associated with sarcopenia-related parameters in middle-aged women identified with class II/III obesity (body mass index [BMI] ≥ 35 kg/m^2^).

**Methods:**

The study included 104 women with severe obesity (40.23 ± 8.49 years) with an average body fat percentage of 52.45 ± 4.14%. Sarcopenia was assessed using total appendicular skeletal muscle mass (ASMM), appendicular skeletal muscle mass index (ASMMI), and appendicular skeletal muscle mass adjusted by BMI (ASMM/BMI) as evaluated using dual energy X-ray absorptiometry (DXA). Hand grip strength (HGS) and HGS adjusted by BMI (HGS/BMI) were evaluated using dynamometry. Functional performance was assessed using the walking speed test (WS). The explanatory variables were age, lifestyle, comorbidities, food consumption, and metabolic parameters. A multivariate linear regression was performed.

**Results:**

Factors associated with sarcopenia-related variables in 104 severely obese women with a mean BMI of 43.85 kg/m^2^ were as follows: ASMMI negatively correlated with serum levels of tetraiodothyronine (T4) and tobacco use; ASMM/BMI negatively correlated with age, serum T4 levels, and diabetes; ASMM negatively correlated with T4 serum levels and diabetes; HGS negatively correlated with age and hypercholesterolemia, and positively correlated with low-density lipoprotein cholesterol (LDL-c); HGS/BMI negatively correlated with age and hypercholesterolemia and positively correlated with LDL-c; and WS negatively correlated with hypothyroidism and diabetes.

**Conclusion:**

In severely obese women, muscle mass and function were inversely associated with age, smoking status, endocrine parameters, hypercholesterolemia, and comorbidities such as diabetes. Thus, the results of this investigation are relevant in supporting the development of clinical interventions to aid in the prevention of sarcopenia in adult women with severe obesity.

## Background

The concept of sarcopenia initially focused only on physiological aging and the decline of skeletal muscle mass [1]. The current evidence suggests that sarcopenia affects both older and younger adults [2]. Mass, strength, and muscular function are considered the main components to consider when making a diagnosis of sarcopenia [3–5]. Present efforts seek to understand the different mechanisms associated with muscle mass reduction and the progressive loss of muscle strength and function [6]. In this context, the presence of obesity, marked by excess adipose tissue, is related to muscle mass loss and contributes to the development of functional and physiological impairments [7–9]. However, there are a variety of propositions and discussions regarding assessment, diagnosis, and cutoff points to define sarcopenia, which hinders accurate comparisons, adequate understanding of the associated factors, and deepening understanding of their relationships with obesity [10, 11].

Obesity is increasing worldwide, and the prevalence of obesity class II (Body Mass Index [BMI] = 35–39.9 kg/m^2^) and III (BMI ≥40 kg/m^2^) has been rising alarmingly faster than that of class I [12]. The highest obesity class is a major public health problem associated with increased health problems and early deaths. Sarcopenic obesity, characterized by simultaneous muscle mass loss and increased body fat, has been recognized as a condition that requires greater clinical attention due to the unfavorable outcomes that can lead to disability, difficulty in carrying out daily activities, and increased mortality [13–15]. Despite the clinical importance of the two simultaneous conditions, obesity and sarcopenia-related parameters, few studies have analyzed this conditions in middle-aged adults [16, 17] and mainly in severely obese individuals [14, 18]. Previous research suggests that poor nutrition is an underlying cause of sarcopenia and that dietary interventions may prevent or treat the loss of muscle mass and strength [6, 19]. However, the clinical, metabolic, and lifestyle factors associated with these parameters have not been fully elucidated [20]. Another point that is important to investigate is the intake of some food sources of antioxidant vitamins, such as vitamins C, D and E, can be associated with muscle mass, functionality, and strength [21, 22].

Understanding the association between sarcopenia-related parameters with several risk factors may help implement interventions aimed at preventing its occurrence by reducing or controlling the identified risk factors [23, 24]. Although changes in body composition occur in both sexes, in women, muscle loss is accentuated due to decreased estrogen levels following menopause [25, 26]. Considering that the adult and elderly population is increasing rapidly worldwide and that women have a higher life expectancy than men, a higher prevalence of obesity and sarcopenia may be observed among women in the future. Therefore, recognizing the factors that contribute to sarcopenia in severely obese young adult women is relevant to directing protective measures to this population group [27]. The results of this study can help clinical obesity treatment settings to prevent sarcopenia and poor health outcomes, such as increasing early mortality Thus, the purpose of this study is to investigate factors associated with the sarcopenia-related parameters in adult women with severe obesity (class II/III).

## Methods

### Study design

This study analyzes baseline data from the clinical trial “Effect of nutritional intervention and olive oil in severe obesity (DieTBra Trial)” [28] (project registered at ClinicalTrials.gov [NCT02463435]). Other results from this multidisciplinary clinical trial have already been published [29–34].

Inclusion criteria were female sex, BMI ≥35 kg/m^2^, age between 18 and 65 years, and residence in the metropolitan area of Goiania, Brazil. Individuals were referred to the Nutrition in Severe Obesity Outpatient Clinic, and the study took place at the Clinical Research Unit of the Clinical Hospital at the Federal University of Goiás. Exclusion criteria were individuals that have had bariatric surgery, pregnant and/or lactating women, physical disability, weight loss ≥8% in the past 3 months, under nutritional/medical follow-up for weight loss for the past 2 years, and current use of anti-obesity drugs. Those who did not meet the specifications for performing body composition tests on the equipment dual energy X-ray absorptiometry (DXA), e.g., those with a pacemaker and/or metal rods and/or screws in the body, were also excluded.

### Sarcopenia-related variables: muscle mass, muscle strength and walking speed test

Muscle mass was evaluated using DXA (GE Healthcare, Lunar DPX NT®, Madison, USA). The half-scanner protocol (hemiscan) was used in patients who were larger than the bed edge limits of the DXA device (> 1.03 m). This protocol includes automatic duplication of the right side to obtain the total body composition. The device was calibrated daily. For the DXA scans, participants were advised to fast for 12 h before the exam; not to perform any physical activity; not to consume energy drinks, coffee, tea, or soda one day before the exam; not to drink alcoholic beverages two days before the exam; and to wear light clothing without metal parts and have the bladder voided during the scan.

The determination of appendicular skeletal muscle mass (ASMM), i.e., the sum of the lean muscle mass of the arms and legs in kg, was performed. ASMM was assessed as total ASMM (kg), adjusted for squared height (appendicular skeletal muscle mass index [ASMMI]) and for BMI (ASMM/BMI) [2, 6].

Hand grip strength (HGS) was assessed to obtain an approximation of the total body muscle strength [35]. The maximum HGS was determined using a hydraulic hand dynamometer (JAMAR®, Hand Dynamometer, Sammons Preston, Inc., Bolingbrook, IL) based on three consecutive measurements with a one-minute interval with the dominant upper limb in the orthostatic position and extended from the body. The maximum HGS value alone and adjusted for BMI (HGS/BMI) were included as outcome variables since they are variables included in the evaluation of sarcopenia [4, 35].

The walking speed test was performed to evaluate the individuals’ functionality [36]. The test was conducted in an unobstructed 6-m-long flat corridor with cones indicating the course start and end points. The course was performed three times at the usual walking speed, with an average interval of one minute between each evaluation. The time required to go through the course was recorded using a digital timer, and the mean value of the obtained measurements was used for the classification [36, 37].

### Anthropometry

Body weight was measured in kilograms using a calibrated digital electronic scale (WELMY©) with a capacity of up to 200 kg and a precision of 100 g. Stature was determined using a stadiometer to the nearest 0.1 cm. BMI was calculated according to the method stated by the World Health Organization (WHO) [38].

### Lifestyle

A trained member of the research team applied a standardized questionnaire to record lifestyle variables including smoking, binge drinking, and physical activity. Smoking status was divided into non-smoker, smoker, and former smoker. The consumption of alcoholic beverages was assessed using an adapted version of the questionnaire used in the study Gender, Alcohol and Culture: An International Study (GENACIS). The binge drinking variable was evaluated based on the consumption of five drinks or more in a single occasion during the last year [39].

Physical activity was evaluated using a triaxial accelerometer (ActiGraph, wGT3X, Pensacola, FL, USA). The accelerometer was used for 24 h a day during six consecutive days over the non-dominant wrist, even during showers and water-based activities, as the device was waterproof. The sampling frequency of the accelerometer was set at 30 Hz, and the data collection interval was set at one min. Accelerometers were set up and downloaded using ActiLife 6 software and data were processed using the R-package, GGIR (http://cran.r-project.org). The outcome measures used in the present study were moderate-to-vigorous physical activity (> 100 milligravities) defined as estimated time spent ≥10 min per bout during a week [40, 41].

### Health conditions and metabolic tests

For the assessment of health conditions, the patients were asked if a medical doctor had ever diagnosed them with a certain disease on a list of diseases of interest for this study, answering yes or no. The presence of the disease was confirmed by the evaluation of the patients’ medication and metabolic tests, as well as blood pressure levels. The health conditions evaluated were diabetes mellitus, hypertension, hypothyroidism, hypercholesterolemia, and menopause.

The presence of diabetes mellitus was verified using the evaluation of the use of hypoglycemic agents and/or fasting blood glucose ≥126 mg/dl and glycosylated hemoglobin (HbA1C) ≥6.5% [42]. The assessment of hypertension occurred based on the mean of two measurements of the systolic (SBP) and diastolic blood pressure (DBP) levels. Patients with SBP ≥140 mmHg and/or DBP ≥90 mmHg or who were using antihypertensive medication were considered hypertensive.

Hypothyroidism was assessed using thyroxine-based drugs and/or serum thyroid stimulating hormone (TSH) > 4.12 μg/l and free tetraiodothyronine (T4) < 0.7 ng/dl [33, 34]. The presence of menopause was investigated based on prior medical diagnosis through the question “Has a physician ever said that you are in menopause?” or the use of hormone replacement therapy. Hypercholesterolemia was defined as low-density lipoprotein cholesterol (LDL-c) ≥160 mg/dl [43].

The metabolic variables analyzed were total cholesterol (TC), high density lipoprotein cholesterol (HDL-c), LDL-c, triglycerides (TG), serum vitamin D, TSH, T4, fasting blood glucose, HbA1C, C-reactive protein (CRP), and parathyroid hormone (PTH). All variables were evaluated according to standardized reference methods [42–46].

### Food consumption

For the evaluation of food consumption, the average nutrient consumption obtained from three 24-h recalls (24HR) was used. The calculation of the average intake of the nutrients of interest was performed using AVANUTRI software. Adequate micronutrient intake was evaluated according to the Estimated Average Requirement (EAR) for adults: vitamin C ≥ 75 mg/day, vitamin D ≥ 10 μg/day, and vitamin E ≥ 12 mg/day [47]. The adequacy of macronutrients was assessed according to the Dietary Reference Intake (DRI) recommended ranges: 10–35% of the total energy intake of proteins and 20–35% of lipids [47].

### Ethical aspects

The HC/UFG Ethics Committee approved the study (protocol 747,792). The larger study was registered at ClinicalTrials.gov (NCT02463435). All participants read and signed the consent inform agreement document to be included in this study.

### Statistical analysis

All statistical analysis was performed using Stata/SE 12.0. The outcomes of this study were the following sarcopenia-related variables: ASMMI (kg/m^2^), ASMM/BMI, total ASMM (kg), HGS, and walking speed. All of them were analyzed as continuous variables. We considered the following explanatory variables: age, lifestyle, health conditions, food consumption, and metabolic parameters. The normality of continuous variables was evaluated using the Kolmogorov-Smirnov test. The descriptive analysis was expressed by mean and standard deviation (SD).

All variables with *p* < 0.20 in the simple linear regression (SLR) were included in the multivariate linear regression model (MLR). In the MLR, variables were adjusted through elimination using the backward method. The level of statistical significance level was set at *p* < 0.05.

## Results

The study included 104 women with severe obesity (mean BMI, 43.85 ± 4.53 kg/m^2^); mean age, 40.23 ± 8.49 years, and average body fat percentage, 52.45 ± 4.14%. The mean and standard deviation of sarcopenia-related variables according to age is shown in Table 1.

**Table 1:** Characterization of the evaluated sarcopenia-related variables according to age group

	Total	18–39 years *N* = 49	≥ 40 years *N* = 55	
Sarcopenia-related parameters	Mean ± SD	Mean ± SD	Mean ± SD	p*
**ASMMI**	8.07 ± 1.15	7.98 ± 1.04	8.14 ± 1.24	0.473
**ASMM/BMI**	0.47 ± 0.09	0.47 ± 0.09	0.45 ± 0.08	0.055
**Total ASMM**	20.34 ± 3.30	20.78 ± 3.42	19.94 ± 3.17	0.201
**HGS (Kgf)**	22.08 ± 6.43	23.02 ± 6.48	21.24 ± 6.33	0.159
**HGS/BMI**	0.51 ± 0.16	0.54 ± 0.16	0.48 ± 0.16	0.083
**Walking speed (m/s)**	1.02 ± 0.18	1.02 ± 0.19	1.01 ± 0.17	0.837

Note. *SD* Standard Deviation, *BMI* Body Mass Index, *ASMMI* Appendicular Skeletal Muscle Mass Index, *ASMM* Total Appendicular Skeletal Muscle Mass, *ASMM/BMI* Total Appendicular Skeletal Muscle Mass/Body Mass Index, *HGS* Hand Grip Strength, *HGS/BMI* Hand Grip Strength/Body Mass Index, *Student’s t-test

The variables included in the MLR of the ASMMI outcome were smoking, menopause, and serum levels of TSH and T4. The variables included in the MLR of ASMM/BMI were age, diabetes, hypertension, menopause, and serum levels of HDL-c, T4 and HbA1C. Finally, the variables included in the MLR for the total ASMM were age, smoking, diabetes, menopause, and serum levels of T4 (Table 2).

**Table 2:** Simple linear regression of the explanatory variables according to different variables related to skeletal muscle mass

	ASMMI		ASMM/BMI		Total ASMM	
	ß	p	ß	P	ß	p
Age	−0.002	0.885	−0.003	**0.008**	−0.070	0.069
Smoking	−0.263	0.158	−0.018	0.210	−0.692	0.197
Binge drinking	− 0.306	0.215	−0.005	0.791	−0.433	0.543
Physical activity	0.003	0.249	< 0.001	0.682	0.006	0.458
Diabetes	−0.229	0.401	−0.057	**0.005**	−0.541	**0.048**
Hypertension	0.116	0.608	−0.026	0.137	−0.079	0.903
Hypothyroidism	−0.188	0.466	−0.009	0.645	−0.408	0.583
Hypercholesterolemia	0.117	0.741	−0.002	0.929	0.130	0.899
Menopause	−0.475	0.111	−0.041	0.068	−0.974	**0.020**
Lipid intake	0.007	0.703	< 0.001	0.831	0.362	0.467
Protein intake	−0.022	0.353	<−0.001	0.763	−0.044	0.515
Vitamin C intake	< 0.001	0.654	< 0.001	0.590	< 0.001	0.621
Vitamin D intake	−0.006	0.257	< 0.001	0.927	0.018	0.218
Vitamin E intake	0.007	0.632	< 0.001	0.955	0.032	0.440
Total cholesterol	−0.003	0.351	<− 0.001	0.640	− 0.004	0.669
HDL-c	−0.010	0.310	−0.001	0.160	−0.037	0.214
LDL-c	−0.002	0.531	<−0.001	0.878	−0.001	0.851
TG	< 0.001	0.932	0.467	0.947	0.001	0.674
Serum vitamin D	−0.002	0.852	< 0.001	0.976	0.002	0.947
TSH	0.114	0.166	<−0.001	0.878	0.213	0.368
T4	−0.295	**0.006**	−0.021	**0.010**	−0.826	**0.007**
Fasting blood glucose	0.002	0.521	<−0.001	0.314	< 0.001	0.940
HbA1C	−0.043	0.596	−0.010	0.111	−0.270	0.253
CRP	0.018	0.425	0.001	0.437	0.048	0.445
PTH	< 0.001	0.962	< 0.001	0.418	0.013	0.307

Note. *BMI* Body Mass Index, *ASMMI* Appendicular Skeletal Muscle Mass Index, *ASMM* Total Appendicular Skeletal Muscle Mass, *ASMM/BMI* Total Appendicular Skeletal Muscle Mass/Body Mass Index

The variables included in the MLR for the HGS outcome included age, excessive drinking, diabetes, hypercholesterolemia, menopause, vitamin C intake, and serum levels of HDL-c, LDL-c, and PTH. In the MLR for walking speed, the included variables comprise aerobic physical activity, diabetes, hypothyroidism, protein intake, and serum TSH and CRP. In the MLR for HGS/BMI, the following variables were included: age, excessive drinking episodes, diabetes, hypertension, hypercholesterolemia, menopause, and serum levels of HDL-c, LDL-c, TG, HbA1C, and PTH (Table 3).

**Table 3:** Simple linear regression of the explanatory variables according to different parameters of strength and walking speed

	HGS		HGS/BMI		Walking speed	
	ß	*p*	ß	*p*	ß	*p*
Age	−0.171	**0.021**	− 0.005	**0.006**	− 0.002	0.392
Smoking	0.658	0.530	0.012	0.642	−0.030	0.301
Binge drinking	0.150	0.147	0.051	0.142	0.037	0.340
Physical activity	−0.010	0.476	−0.001	0.418	0.001	**0.031**
Diabetes	−0.443	0.108	−0.087	0.024	−0.092	**0.031**
Hypertension	− 0.381	0.277	−0.058	0.069	− 0.038	0.283
Hypothyroidism	−0.604	0.677	−0.014	0.702	−0.083	**0.039**
Hypercholesterolemia	−0.322	0.117	−0.078	0.120	0.051	0.358
Menopause	−0.177	**0.025**	−0.084	0.046	−0.041	0.386
Lipid intake	0.035	0.721	−0.001	0.935	−0.001	0.977
Protein intake	0.056	0.672	0.002	0.596	0.005	0.136
Vitamin C intake	−0.003	0.198	−0.001	0.225	−0.001	0.828
Vitamin D intake	−0.013	0.633	0.001	0.852	−0.001	0.217
Vitamin E intake	0.038	0.646	0.178	0.998	−0.001	0.929
Total cholesterol	0.017	0.340	0.001	0.376	−0.001	0.810
HDL-c	−0.093	0.107	−0.002	0.111	−0.001	0.829
LDL-c	0.035	0.056	0.001	0.069	0.001	0.914
TG	−0.011	0.212	−0.001	0.179	−0.001	0.245
Serum vitamin D	−0.057	0.383	−0.001	0.355	−0.001	0.900
TSH	−0.134	0.772	−0.008	0.482	−0.033	**0.008**
T4	−0.074	0.903	−0.005	0.725	0.005	0.751
Fasting blood glucose	−0.011	0.494	−0.001	0.201	−0.001	0.654
HbA1C	−0.544	0.236	−0.018	0.114	0.012	0.348
CRP	−0.033	0.784	−0.001	0.963	0.010	**0.009**
PTH	0.059	**0.015**	0.001	**0.036**	0.001	0.202

Note. *BMI* Body Mass Index, *HGS* Hand Grip Strength, *HGS/BMI* Hand Grip Strength/Body Mass Index

After MLR, ASMMI was negatively associated with serum levels of tetraiodothyronine (T4) and current smoker status. ASMM/BMI and total ASMM were negatively associated with serum T4 levels and the presence of diabetes (*p* = 0.006 and *p* = 0.019). ASMM/BMI was also negatively associated with age (Table 4).

**Table 4:** Multiple linear regression adjusted by the explanatory variables for the different variables related to skeletal muscle mass

	ASMMI		ASMM/BMI		Total ASMM	
	ß (95% CI)	*P*	ß (95% CI)	*p*	ß (95% CI)	*p*
**Age**	–		−0.002 (− 0.004 – − 0.001)	0.043	–	
Smoking*						
Non-smokers	1.00		–		–	
Former smokers	−0.229 (−0.807–0.349)	0.433	–		–	
Smokers	−0.408 (− 0.804 – − 0.012)	0.044	–		–	
**Diabetes**						
No	–		1.00		1.00	
Yes	–		−0.050 (−0.92 – − 0.008)	0.019	−2.488 (−4.230 – − 0.746)	0.006
**T4**	− 0.243 (− 0.328 – − 0.159)	< 0.001	−0.019 (− 0.025 – − 0.013)	< 0.001	−0.674 (− 0.874 – − 0.475)	< 0.001

Note. *BMI* Body Mass Index, *ASMMI* Appendicular Skeletal Muscle Mass Index, *ASMM* Total Appendicular Skeletal Muscle Mass, *ASMM/BMI* Total Appendicular Skeletal Muscle Mass/Body Mass Index, *CI* Confidence Interval

In the MLR model, HGS and HGS/BMI were negatively associated with age, the presence of hypercholesterolemia, and directly associated with serum LDL-c levels (*p* = 0.007). Walking speed was negatively associated with hypothyroidism (*p* = 0.042) and diabetes (*p* = 0.005) (Table 5).

**Table 5:** Multiple linear regression adjusted by the explanatory variables for the variables used in the evaluation of gait force and walking speed. Goiânia, Goiás (Brazil), 2017 (*n* = 104)

	HGS		HGS/BMI		Walking speed	
	ß (95% CI)	*p*	ß (95% CI)	*P*	ß (95% CI)	*p*
**Age**	−0.182 (− 0.358 – − 0.005)	0.043	-0.005 (− 0.010 – − 0.001)	0.016	–	
**Hypercholesterolemia**						
No	1.00		1.00		–	
Yes	−4.918 (−8.725 – −1.112)	0.012	−0.133 (−0.238 – − 0.029)	0.012	–	
**Hypothyroidism**						
No	–		–		1.00	
Yes	–		–		−0.119 (−0.233 – − 0.005)	0.042
**Diabetes**						
No	–		–		1.00	
Yes	–		–		−0.087 (−0.148 – − 0.026)	0.005
**LDL-c**	0.061 (0.017–0.104)	0.007	0.001 (0.001–0.002)	0.031	–	

Note. *BMI* Body Mass Index, *HGS* Hand Grip Strength, *HGS/BMI* Hand Grip Strength/Body Mass Index, *CI* Confidence Interval

## Discussion

To our knowledge, this is the first study to address the factors associated with different sarcopenia-related variables in adult women with severe obesity (class II/III). The assessment of factors associated with muscle mass, strength, and function contribute to the scientific knowledge in the area, mainly due to the wide variety of aspects that may be involved in the determination of the sarcopenia-related variables that were analyzed in the present study. In addition, it is a subject of great importance for women’s health, since they are more affected than men by diseases related to obesity [5, 48].

Among the factors associated with low muscle mass, age, high T4 levels, smoking, and the presence of diabetes are noteworthy. Another relevant finding in our study was that HGS and HGS/BMI reduced with advancing age and the presence of hypercholesterolemia. Additionally, walking speed significantly reduced with the presence of diabetes and hypothyroidism.

The observed association between advancing age and decreased ASMM/BMI, HGS, and HGS/BMI is partly explained by the progressive reduction of anabolism and increased muscle catabolism associated with aging [49]. These events are accompanied by reduced muscle regeneration capacity, an imbalance in muscle protein turnover, and tissue remodeling [49]. The result of this process is the loss and atrophy of individual fibers as well as the loss of fast motor fibers (type II) and a size increase in the slow motor units (type I), with consequent development of muscle weakness and loss of fine movements. Aging-related decreased muscle mass is associated with quantitative and qualitative muscle loss, triggering functional imbalances marked by a decline in strength and functionality [50]. There are no previous studies in adults, obese or not obese, with which to compare the findings of this study. The results highlight the importance of the monitoring of lean mass in the adult population with obesity, since it is intrinsically related to possible disability and limitations.

The observed association between smoking and low ASMMI can be attributed to the influence of tobacco in promoting muscle catabolism [51]. Chronic exposure to cigarette smoke interferes with the signaling of skeletal muscle cells involved in protein homeostasis. These changes are influenced by the duration of exposure and may be reversible following smoking cessation [52]. The mechanisms involved in the low muscle mass of smokers include a reduction in the cross-sectional area of type I fibers, increased glycolytic enzymatic activity, and a decrease in the endothelial and neuronal activities of nitrite oxide synthetase when compared to those in non-smoking controls [53]. Smokers also present with higher levels of tumor necrosis factor alpha (TNF-α), a potent inducer of skeletal muscle protein degradation [54].

Histologically, sarcopenia is defined by muscle fiber atrophy, with high body fat responsible for fatty infiltration into the muscle tissue or lipid deposition within muscle fibers [55, 56]. This occurs in some pathological conditions such as obesity, diabetes, and dyslipidemias [57]. The prevalence of sarcopenia is higher in patients with type 2 diabetes mellitus than in non-diabetic individuals [58]. In this study, diabetes was associated with a decrease in ASMM/BMI, ASMM, and slower walking speed. Such associations can be understood by the fact that skeletal muscle is the main tissue affected by disturbances in glucose metabolism, which, once reduced, triggers a functional decline. Metabolic signaling deficits in this tissue contribute to systemic insulin resistance directly affecting catabolic effects and glucose homeostasis, resulting in lower muscle mass [59, 60].

The association between elevated levels of T4 and reduced muscle mass (ASMMI, ASMM/BMI, and total ASMM) found in our study may be due to the action of thyroid hormones in the maintenance of the balance between anabolism and catabolism in cells and tissues, with the muscle tissue the preferred target for this process [61, 62]. In the present study, an association between decreased functionality (walking speed) and the presence of hypothyroidism was identified. Both hypothyroidism and hyperthyroidism can lead to muscle mass loss and reduced functionality [63]. However, these relationships are not yet sufficiently clear. It is known that hormone treatment restores muscle mass and promotes an improvement in functionality. Thus, the diagnosis and early control of the disease becomes important [64, 65].

The excess of adipose tissue in obesity and presence of fat infiltration in muscle tissue may justify the association between the presence of hypercholesterolemia and the decrease in HGS and HGS/BMI. HGS reduction in sarcopenia is associated with increased serum levels of TC, TG, LDL-c, and decreased HDL-c [66, 67]. Furthermore, increased body adiposity contributes to the onset of different cardiometabolic diseases, such as type 2 diabetes and dyslipidemia. A vicious cycle of muscle loss and ectopic fat accumulation may be associated with the presence of these diseases, through a complex interaction of factors including proinflammatory cytokines, oxidative stress, mitochondrial dysfunction, insulin resistance, among other factors that may be involved in this process. However, without previous studies, it is difficult to draw some parallel [56].

Different clinical outcomes, such as increased risk of developing metabolic syndrome, difficulties in performing daily living activities of and mortality are related to muscle mass and low quality of muscle functions [35, 68, 69]. Recent evidence shows that low quality muscle mass is associated with the development of frailty in obese individuals [70]. The negative effects reported by different studies and our findings, such as a reduction in HGS in the presence of hypercholesterolemia, a decrease in walking speed with hypothyroidism and diabetes occurrence, and a reduction in muscle mass with T4 and diabetes, corroborate the simultaneous occurrence of the loss of muscle quality and negative metabolic outcomes and comorbidities in severely obese adults. Higher fat mass and substantial impairment of muscle mass and quality have interrelated and complex mechanisms with metabolic abnormalities [70]. This issue is relevant in the early diagnosis of and for clinical intervention in sarcopenic obese individuals.

The present study presents a significant contribution to the knowledge of the factors associated with the evaluation of sarcopenia in severely obese individuals, identifying variables with negatively related to muscle mass, strength, and function. There is still no consensus for an adequate evaluation and diagnosis of sarcopenia in adults with severe obesity [6, 14]. Existing cutoff points for the diagnosis of sarcopenia are well defined for the elderly population and may constitute a possible limitation of the present study. However, in our analyses, we did not use cutoff points for the elderly, but average differences from the study population itself to minimize this issue. We emphasize that studies aimed to define cutoffs for adults are vital as sarcopenia has been reported in younger adults. Another possible limitation of our study is the size of the sample analyzed. However, considering the prevalence of severe obesity in the world population, the number of individuals recruited is considerable, given that other previous studies on this subject have a similar sample size to that of our study [14, 18].

## Conclusion

We identified relevant and new risk factors to sarcopenia-related variables in severely obese woman including age, smoking status, endocrine parameters (T4), hypercholesterolemia, and comorbidities such as diabetes and hypothyroidism. All these factors are negatively related to muscle mass, strength, and function. Thus, the results of this investigation are relevant in supporting the development of clinical interventions to aid in the prevention of sarcopenia in adults with severe obesity. Our findings are a relevant contribution to the elaboration of clinical intervention policies aimed at preventing loss of muscle mass and functional impairment, physical disability, decreased strength, and muscular endurance in severely obese women. Additional studies with a longitudinal design are necessary to confirm the results found here.

## Data Availability

The datasets used and/or analyzed during the current study are available from the corresponding author on request.

## Abbreviations

24HR: 24-h recall
ASMM: Appendicular skeletal muscle mass
ASMM/BMI: Appendicular skeletal muscle mass adjusted by BMI
ASMMI: Appendicular skeletal muscle mass index
TC: Cholesterol
CRP: C-reactive protein
DRI: Dietary Reference Intake
EAR: Estimated Average Requirement
DXA: Evaluated by dual energy X-ray absorptiometry
HGS: Hand grip strength
HDL-c: High density lipoprotein cholesterol
PTH: Parathyroid hormone
T4: Tetraiodothyronine
TG: Triglycerides
WS: Walking speed
WHO: World Health Organization
